# Reducing income-related inequalities in care and health: insights from Israel

**DOI:** 10.1186/s13584-015-0037-4

**Published:** 2015-08-11

**Authors:** Cathy Schoen

**Affiliations:** Visiting Fellow NYU Wagner and, Executive Director Commonwealth Fund Council of Economic Advisors, NYU Wagner Graduate School of Public Service, 295 Lafayette St. 2nd Floor, New York City, NY 10012 USA

## Abstract

Amir Shmueli assessed income-related disparities in healthcare and health in Israel, extending earlier studies that focused primarily on education, ethnic or geographic differences. The new analysis finds that the poor are more likely to suffer from an array of chronic conditions, despite higher use of primary care and hospital services. The author suggests that lower use of preventive care, patient behaviors, and lack of adherence to physician recommendations likely contribute to the persistence of health disparities. However, the poor are more likely to work at jobs and live in neighborhoods or housing that put their health at risk. Policies will thus likely need to look beyond medical care to broader social services and workplace issues if the goal is to reduce disparities in disability and heart, lung, mental health and other chronic conditions. If Israeli databases include work and community attributes, it would be useful to include such information to enrich the baseline analysis and to assess the relative efficacy of Ministry of Health and sickness funds initiatives aimed at reducing health disparities.

## Background

From a United States perspective, Amir Shmueli’s analysis of income-related inequalities in health and healthcare in Israel is revealing [1]. Within the US we often attribute health disparities to lack of timely access and affordability, in large part driven by lack of health insurance and gaps in insurance benefits. As the Affordable Care Act begins to extend insurance, cover preventive care in full, and provide substantial subsidies for the poor, the hope is that we will see a substantial reduction in health disparities over time.

The Israeli experience that health disparities persist for some but not all conditions, even when access is more equitable and affordable, provides insight for countries beyond Israel. On a hopeful note, the study found no income-related differences in asthma or cancer. This not the case in the United States where cancer rates and mortality are higher in low-income communities, and asthma rates, especially complications from asthma, are higher among those with low incomes [2].

However, the study finds that significant differences persist for heart, lung, depression, and disability, with notably high income-related disparities for ADL limitations. This is despite that fact that the poor in Israel have higher use of primary care. Not surprisingly, given the higher rates of chronic disease, the Israeli poor also have higher rates of hospital use than those with higher income.

As the author notes, the relationship between poor health and low income is not unidirectional. Individuals and families with higher rates of disability or chronic conditions are less likely to be able to work full time. And family members may need to take time off from work to provide supportive care. At the same time, low-income imposes stress on families and may signal individuals and families who are exposed to health risks at work or in their communities (poor housing, environmental risk, healthy food or exercise options, etc.)

Shmueli concludes that Israel’s health system functions equitably – especially in comparison to several other countries. He then speculates that the key reasons for persistent disparities in health outcomes are the use of preventive care, health behaviors, and compliance with physician orders. The choice of these three reasons suggests remedies from the medical care system and more effective use of medical care services. Selection of these three reasons also suggests that patient behavior is a key concern rather than health system responses/behavior or awareness of underlying health risks leading to the observed poor health.

Disparities may well stem from workplace and community risks that undermine the health of individuals and families with low income. Such “social determinants” of health are known to put lower-income individuals and their families at particularly high health risks [3]. Indeed, the study finding that income-related disparities persist even when access to primary care is readily available and affordable, with particularly wide differences in disability, point to the need to look beyond medical care and personal behaviors to inform initiatives to close gaps.

Analyses of health service use and population health within the United States find “hot spots” for health complications for children or adults; when mapped by very local neighborhoods they reveal adverse living and working conditions [4]. Barriers to improving health may also arise from poor communication between patients and physicians or care teams or lack of safe access (including privacy) to needed care within communities. For example, Revital Gross’s analyses of women’s health and care experiences among diverse vulnerable population in Israel indicated that family relationships, attributes of communities, and less positive interactions or clear communication with physicians could put women at risk [5]. Removing such barriers would require more patient-centered care teams and/or care and support systems within and beyond local communities.

The study finding of significant disparities by income in the prevalence of heart problems, lung problems, diabetes and disability but not asthma or cancer is notable. Without further information on lifetime work, community and health service use, it is impossible to understand factors contributing to the differences across these conditions.

However, one might speculate that ready access to medical care has particular benefits for asthma and cancer in terms of early diagnosis, as well as effective treatment, which could affect prevalence rates. In contrast, disability may arise from work or environmental risks. Although medical care may ease pain or facilitate mobility, it does not remove the income related difference in incidence or prevalence. Similarly, heart disease and congestive heart failure may be the outcome of a lifetime of high negative stress at work or in the community [6]. Medical care may moderate, but not remove, the negative consequences for health, and hence is unlikely to affect disease prevalence.

Further research to asses work and family histories, and their association with disease prevalence, could help inform strategies to reduce gaps by intervening early based on an understanding of risk factors.

## Conclusions

As the Israel Ministry of Health seeks to support initiatives and hold sick funds accountable for reducing disparities, it will be important to consider how broader social policies – for example housing and neighborhood or workplace safety – interact with the people and families at risk [7]. It will also be important to understand the extent to which care teams understand and are responsive to factors contributing to observed health conditions. To the extent that sickness fund data on health status and care use allows more targeted, strategic action, initiatives are more likely to reduce income related disparities over the longer-term.

Similarly, if Israeli national data includes information on work and community attributes, building this into the baseline analysis of income related health disparities would enable future research to assess the relative efficacy of varying initiatives. From a US perspective, this study within Israel provides further evidence that extending health insurance with more equitable access to medical care is a first essential step - with potential early payoffs in asthma and cancer care. However, the study leads to the conclusion – shared with other studies - that more than access to medical care will be needed to reduce health disparities.
